# Mortality of Hemato-Oncologic Patients Admitted to a Pediatric Intensive Care Unit: A Single-Center Experience

**DOI:** 10.3389/fped.2022.795158

**Published:** 2022-07-12

**Authors:** Agnes Pechlaner, Gabriele Kropshofer, Roman Crazzolara, Benjamin Hetzer, Raimund Pechlaner, Gerard Cortina

**Affiliations:** ^1^Department of Paediatrics, Medical University of Innsbruck, Innsbruck, Austria; ^2^Department of Neurology, Medical University of Innsbruck, Innsbruck, Austria

**Keywords:** pediatric intensive care unit (PICU), critically ill children, outcome, trends, hemato-oncology

## Abstract

**Introduction:**

Mortality in children with hemato-oncologic disease admitted to a pediatric intensive care unit (PICU) is higher compared to the general population. The reasons for this fact remain unexplored. The aim of this study was to assess outcomes and trends in hemato-oncologic patients admitted to a PICU, with analytical emphasis on emergency admissions.

**Methods:**

Patients with a hemato-oncologic diagnosis admitted to a tertiary care university hospital PICU between 1 January 2009 and 31 December 2019 were retrospectively analyzed. Additionally, patient mortality 6 months after PICU admission and follow-up mortality until 31 December 2020 were recorded.

**Measurements and Main Results:**

We reviewed a total of 701 PICU admissions of 338 children with hemato-oncologic disease, of which 28.5% were emergency admissions with 200 admissions of 122 patients. Of these, 22 patients died, representing a patient mortality of 18.0% and an admission mortality of 11.0% in this group. Follow-up patient mortality was 25.4% in emergency-admitted children. Multivariable analysis revealed severe neutropenia at admission and invasive mechanical ventilation (IMV) as independent risk factors for PICU death (*p* = 0.029 and *p* = 0.002). The total number of PICU admissions of hemato-oncologic patients rose notably over time, from 44 in 2009 to 125 in 2019.

**Conclusion:**

Although a high proportion of emergency PICU admissions of hemato-oncologic patients required intensive organ support, mortality seemed to be lower than previously reported. Moreover, in this study, total PICU admissions of the respective children rose notably over time.

## Introduction

After trauma, cancer represents the most common cause of death in children and adolescents over 1 year of age in industrialized countries (1). The prognosis of children with cancer has greatly improved in the last decades and overall 5-year survival rate in Europe today exceeds 78.0% (2). Pediatric patients with hemato-oncology frequently face aggressive therapeutic regimes and are at high risk of complications. About 38.0% of these patients have been shown to require intensive care at least once during the course of their disease, and mortality increases dramatically if admission to a pediatric intensive care unit (PICU) becomes necessary (3, 4). Previous studies demonstrated that respiratory failure and sepsis are the predominant reasons for PICU admissions of the respective patients (5, 6). Moreover, recent studies suggest that the outcome of these patients has improved over the years (5, 7, 8). However, a recent meta-analysis including 31 studies in this field suggests that PICU mortality of patients with hemato-oncologic disease has remained relatively unchanged over the past decades (3). Moreover, the authors identified the use of invasive mechanical ventilation (IMV), ionotropic support, or continuous renal replacement therapy (CRRT) as independent risk factors for PICU mortality. In contrast, ICU survival of adult patients with cancer has significantly improved over the past decades (9). The reasons for this unchanged high PICU mortality rate in children with cancer are not fully understood nor studied. Therefore, studies to evaluate PICU outcomes and trends are crucial to improve understanding in the management of this vulnerable subgroup of PICU patients.

The objective of this study was, first, to display an 11-year overview of admission trends of pediatric patients with cancer to a tertiary care PICU and secondly to identify potential risk factors associated with increased PICU mortality in emergency-admitted patients. We hypothesized that stem cell transplantation, severe neutropenia, and the need for organ support such as IMV, ionotropic support, or CRRT would be associated with increased mortality.

## Materials and Methods

### Study Design and Setting

A single-center retrospective study was conducted in a tertiary healthcare center. Consecutive admissions of hemato-oncologic patients transferred to the PICU of the Medical University of Innsbruck, Austria, between 1 January 2009 and 31 December 2019 were analyzed. Innsbruck PICU is an 11-bed multidisciplinary referral institution for western Austria with ~600 admissions per year. The pediatric hemato-oncology department is the second largest in Austria, accounting for up to 80 new cancer diagnoses yearly. This study was approved by the institutional ethical review board [Reference No. 1420/2020].

### Data Collection

Data were collected retrospectively from hospital records. Data collected at admission included basic demographic characteristics such as age, weight, gender, reason for admission, and consecutive number of admissions, as well as underlying hemato-oncologic diagnosis, transplant history, and the presence of severe neutropenia at admission. Regarding ICU details, we collected data on PICU length of stay (LOS), number of organ system failures, PICU mortality, and the cause of PICU death as well as the use of PICU resources such as need and length of IMV, amount and duration of vasopressor or inotropic support, need for CRRT, and/or extracorporeal membrane oxygenation (ECMO). Additionally, mortality 6 months after PICU admission and follow-up mortality until 31 December 2020 were recorded.

Given the differences in the reason for admission (e.g., vital indication vs. postoperative monitoring), all admissions were categorized in three groups: *emergency* comprises all cases of non-elective admissions for organ support due to potentially life-threatening complications; *monitoring* includes admissions following surgery or imaging with subsequent need for intensive care surveillance. *Interventions* contains patients admitted for procedures, e.g., bone marrow puncture requiring sedation in patients at high risk of adverse events, or central venous catheter placement or to prevent/anticipate adverse events in patients, for example, with a mediastinal mass or receiving high-risk infusions (monoclonal antibodies).

Diagnoses were grouped to enable analysis as follows: *Leukemia* describes all patients diagnosed with acute myeloid leukemia (AML), acute lymphatic leukemia (ALL), chronic myeloid leukemia, and acute bilinear leukemia. *Lymphoma* admissions codify for an underlying disease such as B- or T-cell non-Hodgkin lymphoma, Hodgkin lymphoma, anaplastic lymphoma, large granulocyte lymphoma, and post-transplant lymphoproliferative disease (PTLD). *Brain* includes all cases of primary tumors of the central nervous system. *Solid* gathers admissions with diagnoses such as Wilms' tumor, hepatic tumors, neuroblastoma, retinoblastoma, and bone or soft tissue tumors. The *Hematological* group contains all patients with hematological diseases admitted to PICU.

### Definitions

*Severe neutropenia* was defined as an absolute neutrophil count of <500/μl at PICU admission. *Multiple organ dysfunction syndrome (MODS)* was defined as dysfunction of three or more organ systems during PICU stay. MODS and sepsis were codified in accordance with the Pediatric Sepsis Consensus Conference definitions (10).

*Inotropic support* was defined as the administration of one or more of the following vasopressor or inotropic agents: epinephrine, norepinephrine, dopamine, dobutamine, milrinone, or vasopressin. The number of inotropes was defined as the maximum number of inotropes used during the PICU stay. It was quantified by the vasoactive inotropic score (VIS), which was calculated by the formula: 1× [dopamine + dobutamine (mcg/kg/min)] + 10× milrinone (mcg/kg/min) + 100× [epinephrine + norepinephrine (mcg/kg/min)] + 10,000× vasopressin (U/kg/h). Maximal VIS during the PICU stay was recorded.

### Statistical Analysis

The analysis was performed with R version 4.1.1 [R Foundation for Statistical Computing, Vienna, Austria].

The results are presented as median and interquartile range (IQR) or count and percentage, as appropriate. Chi-squared test and Fisher's exact test were used for univariate analysis of categorical data and Wilcoxon rank sum test and logistic regression analysis for continuous data. Multivariable logistic regression was performed to test for potential risk factors for PICU death in emergency-admitted patients. Candidate variables were literature-proven risk factors for PICU death such as history of allogeneic stem cell transplantation (SCT), intensive care therapies, and MODS. MODS was excluded from the model due to its redundancy with the need for IMV, CRRT, and inotropic support. ECMO therapy was only rarely applied and was therefore not considered. We used L1-regularized logistic regression (11) (LASSO) to identify multivariable predictors of PICU mortality in the setting of sparse data (e.g., small sample size of non-survivors) out of the candidate variables severe neutropenia on admission, IMV, inotropic support, CRRT, and history of allogeneic SCT, using leave-one-out cross-validation for selection of the regularization hyperparameter lambda that minimizes deviance. A conventional logistic regression model was then fitted using the variables selected by LASSO to obtain confidence intervals (CIs) and *p*-values. A simple linear regression model was fitted to investigate changes in PICU admission numbers as well as mortality trends in emergency admissions per year. Long-term survival probabilities were estimated with Kaplan–Meier curves and compared with the log rank test. A two-tailed value of *p* < 0.05 was considered statistically significant.

## Results

### Patient Selection

There were 701 PICU admissions of 338 children with hemato-oncological disease, which were categorized into emergencies, monitoring, and interventions. Most patients were admitted for perioperative monitoring (*n* = 271, 38.7%), followed by interventions (*n* = 230, 32.8%) and emergency admissions (*n* = 200, 28.5%). Detailed information on all admissions is found in Supplementary Table S1.

During the study period, we had a total of 4,834 admissions to our PICU. Patients with hemato-oncology accounted for 14.7% of all PICU admissions and for 20.7% of all PICU deaths (24 of 116) during the study period. Moreover, during this period, the PICU mortality rate for hemato-oncologic admissions was higher than the mortality rate for all non-cancer admissions (3.4 vs. 2.2%).

The total number of admissions of patients with hemato-oncology rose significantly over time (*p* < 0.001, β = 9.2, CI 6.2–12.3), from 44 in 2009 to 125 in 2019 (Supplementary Figure S1), accounting for 10.9% of PICU admissions in 2009 (44 of 423) and 19.9% in 2019 (125 of 619).

**Figure 1:**
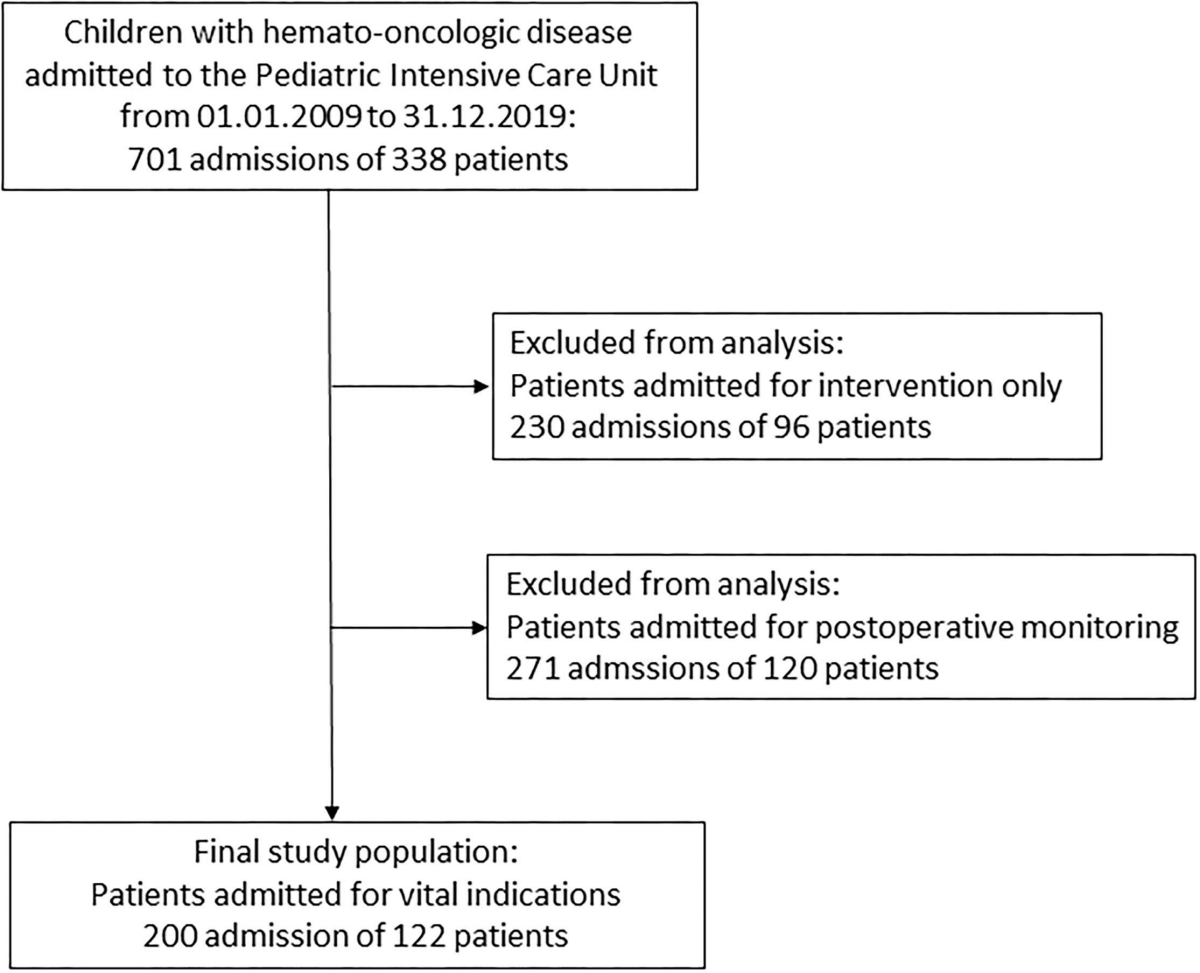
Flow chart of study population.

To identify potential risk factors associated with increased PICU mortality, we excluded patients admitted for interventions and for postoperative monitoring and performed detailed statistics in the emergency patient group (Figure 1).

### Emergency Admissions

During the study period, a total of 122 patients were admitted on 200 occasions for vital indications. The predominant illness was leukemia with 38.0% of all admission diagnoses. The most common reasons for emergency admission were respiratory failure and sepsis with 36.5 and 20% (Supplementary Table S2). The median age at admission was 8.3 years (IQR 3.1–14.7) and median LOS was 2.8 days (IQR 1–7). Emergency admissions ranged between 1 and 10 with a median of one per patient. In total, 82 patients (67.2%) were admitted only once for vital indications during the study period. The number of emergency admission per year increased significantly over time (*p* = 0.002; β = 2.1; CI 1.0–3.2).

Table 1 shows detailed information on emergencies' characteristics and risk factor analysis.

**Table 1:** Characteristics of emergency admissions: survivors vs. non-survivors.

**Characteristic**	**N (%)**			**Estimated differences** **OR (95% CI)**	***p***
	**All admissions** **(*n* = 200)**	**Survivors** **(*n* = 178)**	**Non-survivors** **(*n* = 22)**		
Male gender	102 (51)	90 (50.6)	12 (54.5)	1.2 (0.4–3.2)	0.9
Age^#^, years	8.3 (3.1–14.7)	7.8 (3–8.4)	12.6 (4–15.3)	1.1 (0–0.1)	0.19
^#^PICU length of stay (days)	2.8 (1–7)	2 (1–5.8)	8.5 (4.3–26.5)	1.04 (0–0.1)	**0.006**
Severe neutropenia^†^	62 (31)	49 (27.5)	13 (59)	3.8 (1.4–10.7)	**0.006**
Readmittance	118 (59)	104 (58.4)	14 (63.3)	1.2 (0.5–3.6)	0.82
MODS present	36 (18)	18 (10.1)	18 (81.8)	38.3 (11.1–173.1)	**<0.001**
**Diagnoses**					
Leukemia	76 (38)	65 (36.5)	11 (50)	1.7 (0.6–4.7)	0.32
Lymphoma	29 (14.5)	25 (14)	4	1.4 (0.3–4.6)	0.53
Brain/spinal cord	31 (15.5)	27 (15.2)	4	1.2 (0.3–4.2)	0.76
Solid	28 (14)	28 (15.7)	0	0 (0–1.1)	0.05
Hematological	36 (18)	33 (18.5)	3	0.7 (0.1–2.6)	0.77
**Transplant history**					
Autologous HSCT	15 (7.5)	14 (7.9)	1	0.6 (0–4.1)	1
Allogeneic hSCT	56 (28)	45 (25.3)	11 (50)	2.9 (1.1–8.1)	**0.022**
SOT	8 (4)	8 (4.5)	0	0 (0–4.9)	0.6
None	121 (60.5)	111 (62.4)	10 (45.5)	0.5 (0.2–1.4)	0.165
**PICU treatment**					
IMV	69 (34.5)	49 (27.5)	20 (90.1)	25.9 (5.9–235.5)	**<0.001**
IMV >2 days	46 (23)	29 (16.3)	17 (77.3)	17.1 (5.5–64.1)	**<0.001**
CRRT	18 (9)	11 (6.2)	7 (31.8)	6.8 (0.9–43.3)	**0.03**
ECMO	7 (3.5)	4	3	7 (2–23.4)	**0.001**
Inotropic support	43 (21.5)	28 (15.7)	15 (68.2)	11.3 (3.9–35.9)	**<0.001**
VIS^#^	0 (0–0)	0 (0–0)	22 (0–43.9)	1.1 (0.1–0.2)	**<0.001**

# *Median [interquartile range (IQR)]; PICU, pediatric intensive care unit;*

† *at admission; MODS, multiple organ dysfunction syndrome; HSCT, hematopoietic stem cell transplantation; SOT, solid organ transplantation; IMV, invasive mechanical ventilation; CRRT, continuous renal replacement therapy; ECMO, extracorporeal membrane oxygenation; VIS, vasoactive ionotropic score; OR, odds ratio; CI, confidence interval. Bold values denote statistically significant*.

In total, 22 children died after emergency admission to PICU, leading to a patient mortality of 18.0% and an admission mortality of 11.0% in this group. Admission mortality was reduced to 8.4% if post-HSCT admissions were excluded (*p* = 0.6). Moreover, about every fourth child was reported dead at the follow-up evaluation (31 children, 25.4%).

Over time, no significant decrease in PICU mortality was found in emergency admissions (*p* = 0.62). However, a significant trend toward reduced mortality was seen in the second half of the study period (2014–2019, *p* = 0.009, β = −0.021, CI −0.03 to −0.009).

No underlying diagnosis was associated with higher PICU mortality in the univariate analysis.

The highest admission mortality depending on the reason for emergency admission was seen with 30.0% in patients admitted for acute graft vs. host disease (3 of 10). Sepsis with neutropenia and febrile neutropenia without sepsis were much more common reasons for admission and showed admission mortalities of 18.8 and 16.7% (Supplementary Table S2).

### Mortality and Transplant History

**Figure 2:**
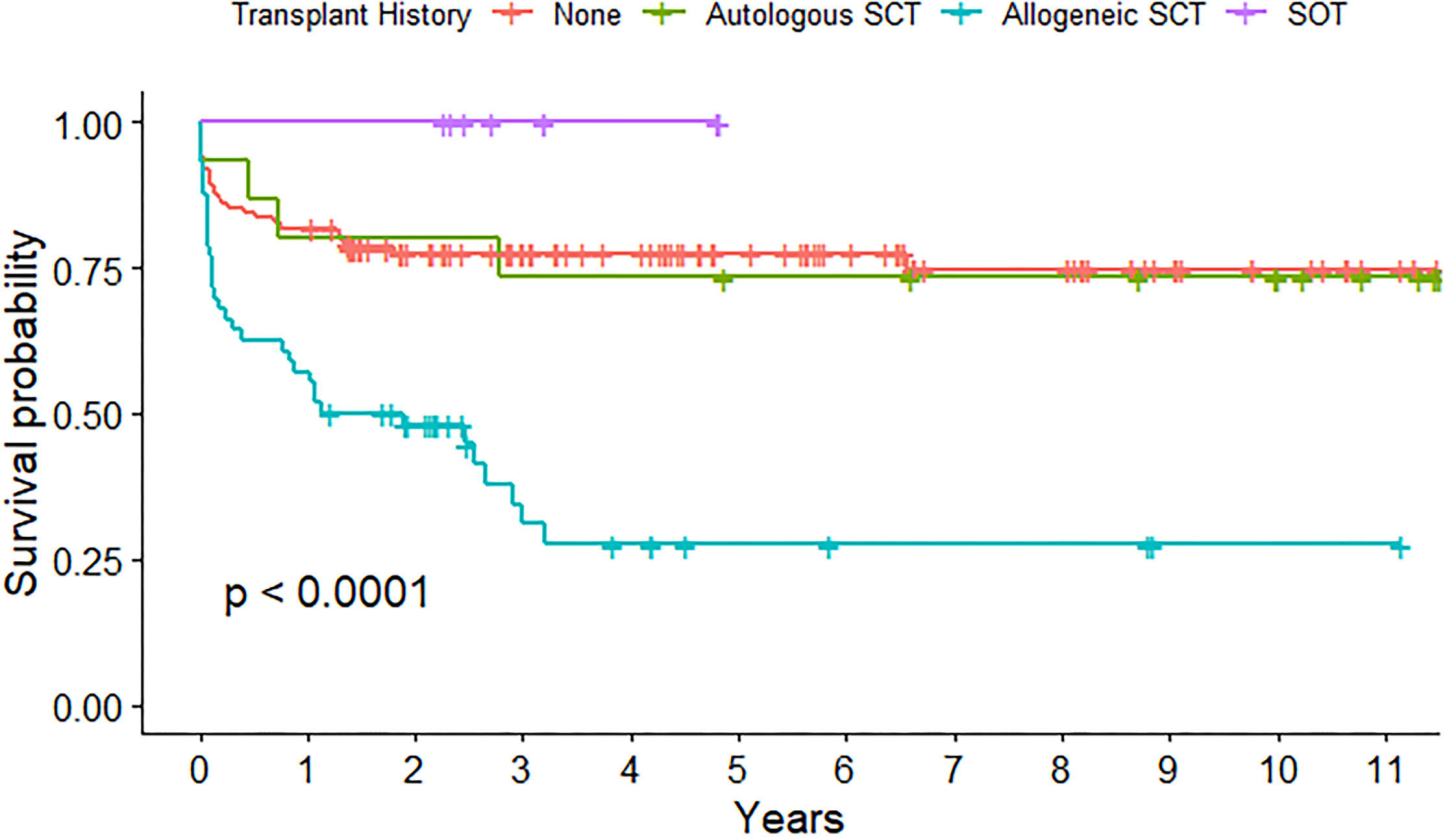
Long-term survival probability after emergency admission to pediatric intensive care unit (PICU) depending on transplant history.

A transplant history was present in 79 admissions, with 15 autologous SCT, 56 allogeneic SCT, and 8 solid organ transplantations (SOT, Table 1). The highest mortality rate was found in patients with a history of allogeneic SCT at admission (11 of 56, 19.6%) and this showed to be a risk factor for PICU death in univariate analysis (*p* = 0.022) but not in multivariable analysis (*p* = 0.08). Long-term survival probability after emergency admission to the PICU was significantly decreased in children with a history of allogeneic SCT (*p* < 0.001), while survival probability of patients with post-SOT was shown to be excellent (Figure 2).

### Mortality and Severe Neutropenia

About one-third of emergency admissions occurred with severe neutropenia (31%) which, when present on admission, represented an independent risk factor for PICU death in the multivariable analysis (*p* = 0.029; OR 3.7, 95% CI 1.2–12.5, **Table 3**).

Multivariable predictors of PICU mortality are shown in Table 2.

**Table 2:** Multivariable predictors of PICU death.

**Variables**	**OR**	**95% CI**	***p***
Allogeneic SCT	2.6	0.9–8.3	0.085
Severe neutropenia at admission	3.7	1.2–12.5	0.029^*^
IMV	15.7	3.3–117.1	0.002^*^
CRRT	2.2	0.6–7.9	0.233
Inotropic support	1.7	0.5–6.5	0.416

*OR, Odds Ratio; CI, Confidence Interval; SCT, stem cell transplantation; IMV, invasive mechanical ventilation; CRRT, continuous renal replacement therapy.*

* *p < 0.05. Predictors were selected using the least absolute shrinkage and selection operator (LASSO). Bold values denote statistically significant*.

### Mortality and Organ Failure

Children who experienced MODS showed an admission mortality of 50.0% (Supplementary Table S3). As expected, MODS was significantly associated with PICU death in univariate analysis (*p* < 0.001; Table 1). If only one or two organ systems failed, admission mortality was 3.7%.

### Use of PICU Resources

Of all emergency admissions, 34.5% required IMV and 23.0% did so for more than 2 days (Table 3). In total, 20 patients with IMV did not survive to PICU discharge (29%). If IMV was the only ICU treatment necessary, PICU mortality was still at 17.9%. Median IMV duration was 4 days (IQR 1–12) and showed to be significantly shorter in survivors than in non-survivors (3 vs. 6.5 days; *p* = 0.048). If both IMV and inotropic support were needed, admission mortality rose to 39.5%. Seven patients received ECMO therapy because of respiratory failure and three did not survive (42.9%). Three patients undergoing ECMO had a history of allogeneic SCT, of whom two survived to PICU discharge. The median length of ECMO usage was 12 days (IQR 4.5–15.5) with survivors receiving longer ECMO support than non-survivors (median ECMO time 15.5 vs. 4 days, *p* = 0.004). Inotropic support was provided in 43 cases (21.5%) with 16 admissions receiving more than two agents during the PICU stay. Survival was inversely related to the number of drugs administered (Supplementary Table S4). Median maximum VIS showed to be significantly lower in survivors than in non-survivors (0 vs. 22, *p* < 0.001; 95% CI 9.3–28.2; Supplementary Figure S2). All admissions requiring CRRT without IMV survived to PICU discharge, but mortality increased to 53.8% if both were needed. When testing PICU resources in multivariable analysis, IMV represented an independent risk factor for PICU death (*p* = 0.002; OR 15.7, 95% CI 3.3–117.1).

**Table 3:** Admission mortality depending on the PICU treatment needed.

**PICU treatment**	***N* (%)**	**Died in PICU**	**Mortality (%)**
Inotropic support alone	5 (2.5)	0	0
CRRT alone	5 (2.5)	0	0
IMV	69 (34.5)	20	29
IMV alone	28 (14)	5	17.9
IMV >2 days	46 (23)	17	40
IMV + Inotropic support	38 (19)	15	39.5
IMV + CRRT	13 (6.5)	7	53.8
ECMO	7 (3.5)	3	42.9

*PICU, pediatric intensive care unit; IMV, invasive mechanical ventilation; CRRT, continuous renal replacement therapy; ECMO, extracorporeal membrane oxygenation*.

## Discussion

The present study adds to the scarce literature on pediatric hemato-oncologic patients admitted to a PICU and its purpose was to identify risk factors associated with increased PICU mortality in emergency-admitted children. Encouragingly, we found that overall mortality and mortality per admission in emergency-admitted patients with cancer were lower than previously reported (6–8, 12), despite the inclusion of post-HSCT admissions in our analyses.

Even a recent meta-analysis including 31 similar studies (3) showed a higher pooled PICU mortality rate at 27.8 vs. 11% admission and 18.0% patient mortality in our analyses of emergency-admitted patients. Wösten-van Asperen et al. noted that the comparability of the included studies was limited due to a strong heterogeneity of inclusion and exclusion criteria, which could partially explain the difference in mortality rates.

The dilemma of comparability of patients and admissions seems omnipresent and frequently lacked distinction in previous research. Unlike Pillon et al. who chose to analyze only the first admission if the patient was readmitted more than 24 h after the first PICU stay (6), we decided to present patient mortality (per individual) and admission mortality (per admission) in accordance with previous research (8). We were aware of its inclination for initial confusion. However, regarding our observed range of 1–16 admissions per patient, we find this method inevitable to ensure precise results.

Regarding the use of PICU resources, we found comparable rates of organ support therapies as the meta-analysis of Wösten-van Asperen, with a high incidence of patients requiring IMV (34.5 vs. 30%), ionotropic support (21.5 vs. 40%), and CRRT (9 vs.4.5%). Moreover, our results identified similar risk factors for PICU death in emergency-admitted patients as previously reported, with slight differences in terms of analytical significance. IMV still represented a significant predictor of higher PICU mortality as already stated before (3, 7, 12, 13). However, contrary to foregone research, in this study both inotropic support and CRRT were not independently associated with higher mortality (3, 14), although mortality was high if inotropic support or CRRT were used in combination to IMV. Most of these patients will experience MODS and this is also reflected in the fact that patients with MODS had a high mortality, whereas patients with only one or two organ systems failures were shown to have a very low mortality.

Improvement in the outcome of hemato-oncologic patients in PICU might be due to improvement in intensive care therapies, such as timely completion of the sepsis treatment bundle (antibiotic and fluid administration, blood cultures), lung protective ventilation strategies, and early use of invasive extracorporeal therapies such as CRRT and ECMO. Patients with hemato-oncologic disease, and especially patients post-HSCT, are at high risk of acute kidney injury and significant fluid overload (FO) (15). CRRT has been shown to be an independent risk factor of PICU mortality and patients with oncology requiring CRRT have been reported to have mortality rates of up to 80% (14). Not surprisingly, most of these patients had MODS and significant FO. The prevention of FO in the first place and early initiation of CRRT to avoid significant FO in these high-risk patients may be the key to improved outcome.

Extracorporeal membrane oxygenation has traditionally been considered as a relative contraindication in patients with hemato-oncologic disease due to higher complication rates and poor prognosis. However, recent research in this field could show reasonable outcome for patients with hemato-oncologic disease treated with ECMO. A case series from our institution showed that 44.4% (four of nine) of children with leukemia survived long-term with good quality of life after requiring ECMO for respiratory failure (16). Two larger ELSO database studies showed similar encouraging results, even in patients who were post-HSCT (17, 18). This might led to the fact that more institution offer extracorporeal therapies to deteriorating hemato-oncologic patients in PICU.

Patients following HSCT have had poor outcomes when admitted to PICU, although in this delicate population the prognosis has improved impressively over the past three decades from 85.0 to 44.0% (19–21). This higher mortality in patients with HSCT compared to patients with non-HSCT oncology made many investigators to exclude these patients from their analyses. Recently, the large North American virtual PICU system database even reported a much lower mortality of 16.2% among 1,782 admission of patients younger than 21 years following HSCT, which is comparable to the 19.6% mortality on admission that we found in our cohort of emergency-admitted patients post-allogeneic HSCT (21). Not surprisingly, similar to our study, invasive ventilation was identified as an independent risk factor for death.

In addition to improved intensive care, multidisciplinary care of critically ill cancer patients involving hemato-oncologists has been shown to improve outcomes (22). Therefore, close collaboration between pediatric intensivists, hemato-oncologists, and infectious disease specialists is essential for early identification of deteriorating patients with cancer on the oncology ward who might profit from early aggressive medical intervention before irreversible organ damage occurs. Additionally, introduction of rapid response teams and development of early warning signs have become novel strategies to assist the haemato-oncology team in decision-making about early transfer of patients with deteriorating to ICU (23, 24). Recently, the European Society for Pediatric and Neonatal Intensive Care (ESPNIC) has created the PICU Oncology Kids in Europe Research Group (POKER) to further intensify collaboration between intensivists and pediatric oncologists. Given the lack of multicenter outcome data, this working group will hopefully help to gather information and to standardize pediatric onco-critical care, which might ultimately lead to better outcomes in critically ill patients with hemato-oncology (25).

Interestingly, in this study, a significant increase in both all hemato-oncologic as well as emergency PICU admissions was noted. This trend may be seen in other institutions as patient safety protocols have become increasingly important. In our institution, after the introduction of early warning signs to identify patients with deterioration, PICU admission practices changed to a more pre-emptive approach and children were admitted earlier in their course of illness. Moreover, children at high risk for adverse events, such as patients at risk for tumor lysis syndrome, with mediastinal mass, or patients receiving novel immunotherapy agents like blinatumomab for ALL, which have been associated with neurological toxicities and cytokine release syndrome (26), have been admitted to our ICU to anticipate potential major problems.

The overall mortality of emergency admissions per year did not decrease in our analyses; however, in the second half of the study period, a trend to mortality reduction could be seen. This could likely be because of more pre-emptive PICU admission practices leading to higher emergency admission numbers while the absolute number of PICU deaths remained relatively unchanged over time.

This study has several strengths. First, it offers up-to-date information of a large sample size of respective patients in central Europe. Its observation period exceeds that of most alike studies known to the authors and it offers at least 1 year of follow-up information on the survival of each included patient. Furthermore, this study population has not been characterized before on a national basis in Austria. Analytical emphasis was put on emergency admissions, but general information on all admissions was also provided. Most importantly, a great effort was made to amend the lack of comparability of previous studies due to unclear inclusion and exclusion criteria, providing patient- and admission-related mortality. Differentiation between patients with SCT and non-SCT was also granted to reduce biased results.

The findings of this survey are limited due to its retrospective nature and single-center approach. An important limitation of this study is that we did not use severity of illness scores at admission, which limits the comparability of our findings with the literature. Although our patients had high rates of organ support therapies, the lower mortality rates, especially in the second half of the study, have to be questioned and it is possible that the lower severity of illness on admission is responsible for the lower mortality rates. As mentioned above, recently, patient safety protocols led to increased PICU admission of patients with oncology. It is possible that we may have admitted fewer sick patients than described in older cohorts and/or that these patients are admitted earlier in their course of illness and benefitted from early therapies such as fluid resuscitation and non-invasive ventilation and did not progress to more severe stages of disease. Therefore, our results, including PICU admission practices, might not be representative of other centers.

## Conclusion

Although a high proportion of emergency PICU admissions of hemato-oncologic patients required intensive organ support, in this study, mortality seemed to be lower than previously reported. Consistent with previous research, severe neutropenia at admission and IMV were identified as independent risk factors for PICU death. Moreover, PICU admissions of the respective children rose over time. Given the importance of this topic, multicenter collaborations are urgently needed to gather data and increase our understanding of this vulnerable patient group.

## Data Availability Statement

The raw data supporting the conclusion of this article will be made available by the authors upon reasonable request.

## Ethics Statement

The studies involving human participants were reviewed and approved by Ethics Committee of the Medical University of Innsbruck. Written informed consent from the participants' legal guardian/next of kin was not required to participate in this study in accordance with the national legislation and the institutional requirements.

## Author Contributions

GC, GK, and RC conceptualized the design of the study. AP, GC, and BH collected the data. AP and RP performed statistical analysis. AP and GC wrote the first draft of the manuscript. All authors carefully edited and corrected the various versions of the manuscript and accepted the final manuscript.

## Conflict of Interest

The authors declare that the research was conducted in the absence of any commercial or financial relationships that could be construed as a potential conflict of interest.

## Publisher's Note

All claims expressed in this article are solely those of the authors and do not necessarily represent those of their affiliated organizations, or those of the publisher, the editors and the reviewers. Any product that may be evaluated in this article, or claim that may be made by its manufacturer, is not guaranteed or endorsed by the publisher.

## Supplementary Material

Supplementary Figure S1 Number of admissions per category and year (*n* = 701). SCT, stem cell transplantation; SOT, solid organ transplantation.

Supplementary Figure S2 Median maximum VIS Score in survivors compared to non-survivors.

Supplementary Table S1 General characteristics of patients and admissions.

Supplementary Table S2 Admission mortality according to reason for emergency admission (*N* = 200).

Supplementary Table S3 PICU mortality according to the number of organ system failures.

Supplementary Table S4 PICU mortality and inotropic support.
