# Supplementary figures and images for: Mortality of Hemato-Oncologic Patients Admitted to a Pediatric Intensive Care Unit: A Single-Center Experience
Source: Front Pediatr. 2022 Jul 12;10:795158. doi: 10.3389/fped.2022.795158 (PMC9315049; doi:10.3389/fped.2022.795158)

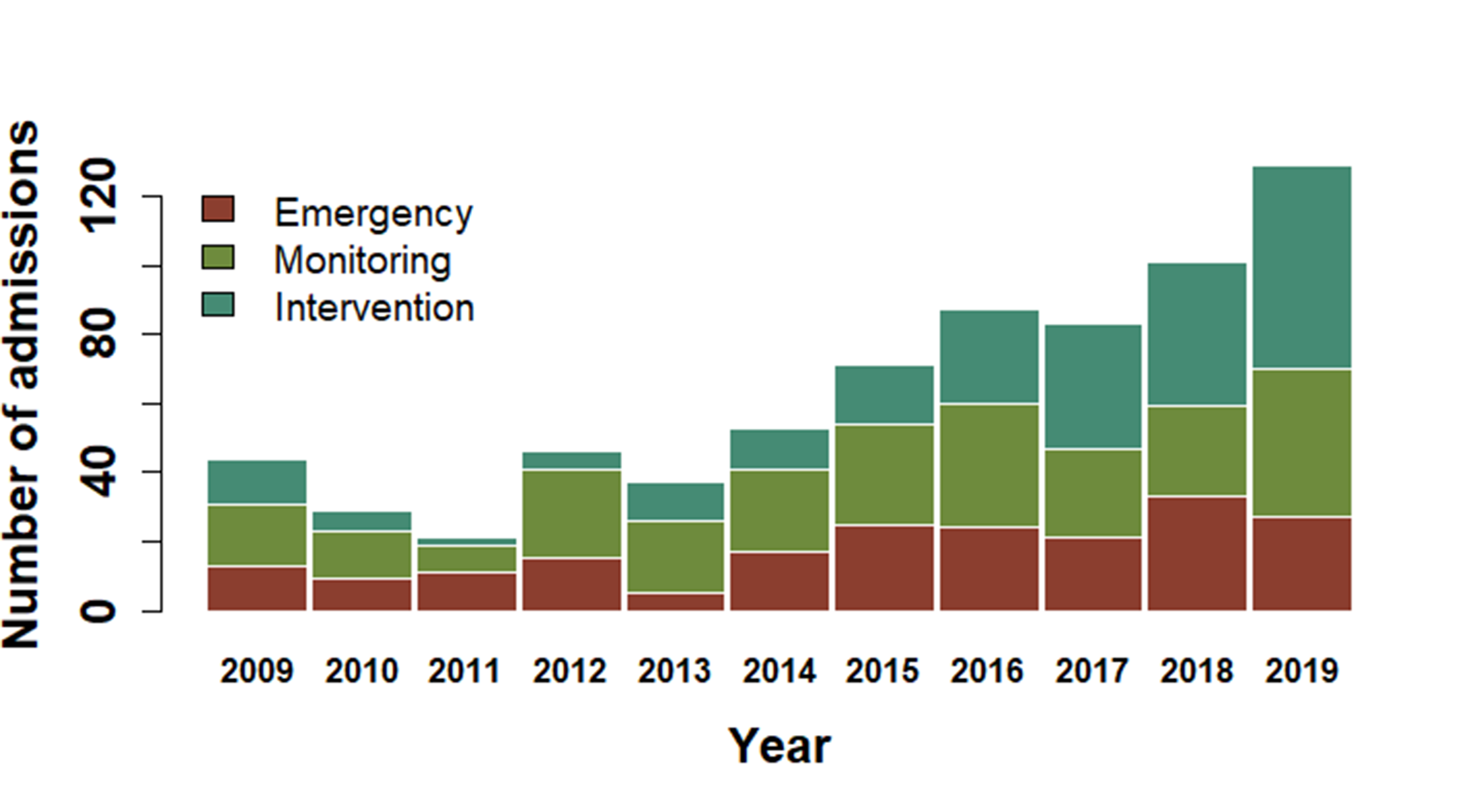

Supplement: Supplementary Figure S1 — Number of admissions per category and year (n = 701). SCT, stem cell transplantation; SOT, solid organ transplantation. [file Image_1.TIFF]

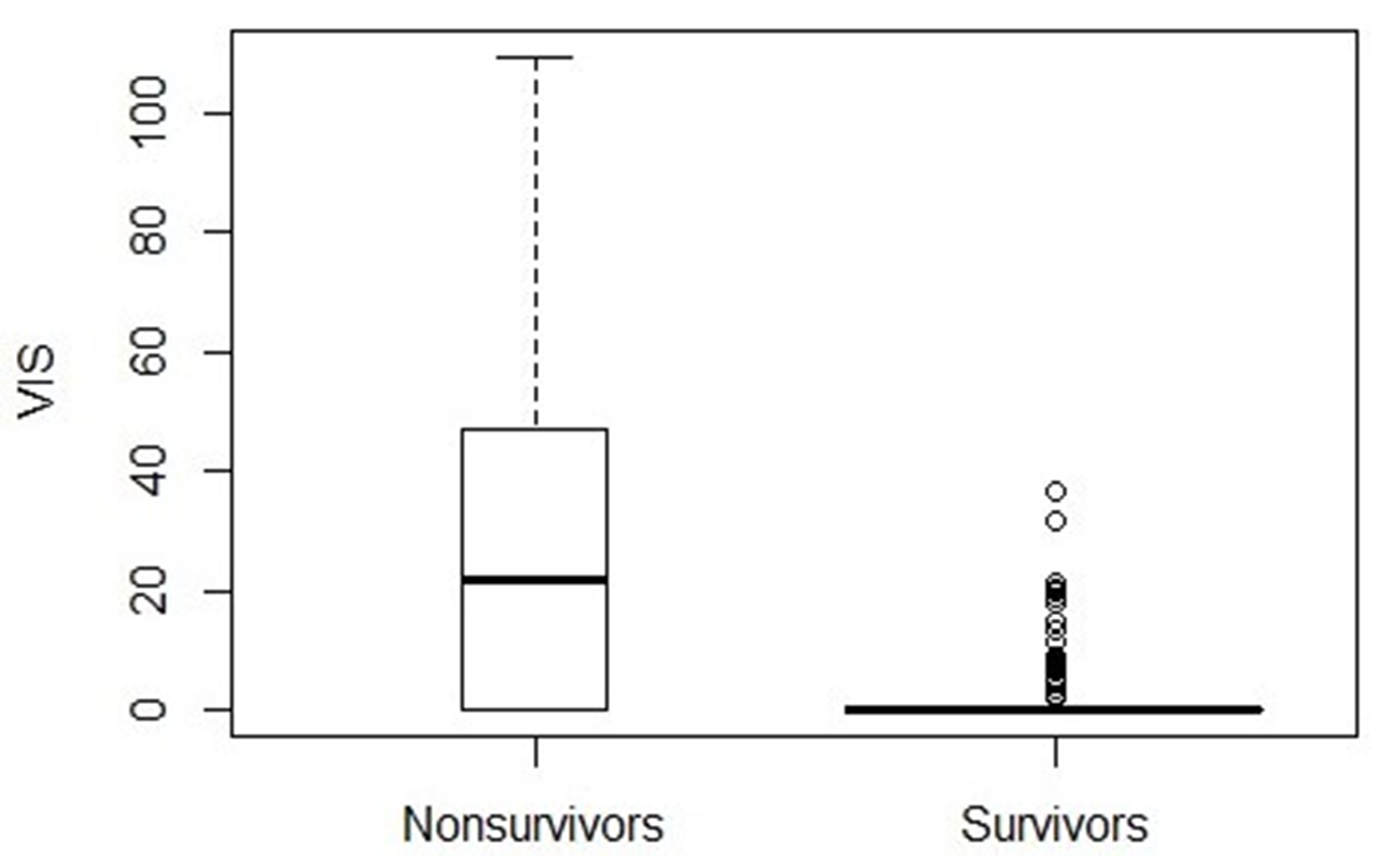

Supplement: Supplementary Figure S2 — Median maximum VIS Score in survivors compared to non-survivors. [file Image_2.TIFF]
