# Supplementary material for: Mortality of Hemato-Oncologic Patients Admitted to a Pediatric Intensive Care Unit: A Single-Center Experience
Source: Front Pediatr. 2022 Jul 12;10:795158. doi: 10.3389/fped.2022.795158 (PMC9315049; doi:10.3389/fped.2022.795158)
Supplement: Supplementary Table S1 — General characteristics of patients and admissions. [file Table_1.DOCX]

**Supplemental Table 1**: General characteristics of patients and admissions

|  | | **Patients***  **(N=338)** | **Admissions (N=701)** | | |
| --- | --- | --- | --- | --- | --- |
|  |  |  | **Emergency (n=200)** | **Monitoring**  **(n=271)** | **Interventions**  **(n=230)** |
|  | |  | n (%) |  |  |
| Male gender | | 186 | 102 | 123 | 120 |
| Age^#^, years | | 7.5 (2.8 – 12) | 8.3 (3.1 – 14.7) | 5.6 (2.1 – 10.3) | 5.9 (2.6 – 11.2) |
| ^#^PICU length of stay (days) | | 1 (0.2 – 2) | 2.8 (1 – 7) | 1 (1 – 2) | 0.2 (0.1 – 0.3) |
| PICU mortality | | 24 (7.1) | 22 (11) | 0 (0) | 2 (0.9) |
| Severe neutropenia† | | 23 (6.8) | 62 (31) | 5 (1.8) | 10 (4.3) |
| *Diagnoses* | |  | | | |
|  | Leukaemia | 84 (24.9) | 76 (38) | 5 (1.8) | 112 (48.7) |
|  | Lymphoma | 25 (7.4) | 29 (14.5) | 5 (1.8) | 19 (8.3) |
|  | Brain/spinal cord | 99 (29.3) | 31 (15.5) | 154 (56.8) | 7 (3) |
|  | Solid | 97 (28.7) | 28 (14) | 101 (37.3) | 60 (26.1) |
|  | Haematological | 33 (9.8) | 36 (18) | 6 (2.2) | 32 (13.9) |
| *Transplant History* | | | | | |
|  | Autologous HSCT | 17 (5) | 15 (7.5) | 32 (11.8) | 9 (3.9) |
|  | Allogeneic HSCT | 22 (6.5) | 56 (28) | 6 (2.2) | 28 (12.2) |
|  | SOT | 7 (2.1) | 8 (4) | 6 (2.2) | 2 (1) |
|  | None | 292 (86.4) | 121 (60.5) | 227 (83.8) | 191 (83) |
| *PICU resource use* | | | | | |
|  | IMV | 147 (34.5) | 69 (34.5) | 209 (77.1) | 7 (3) |
|  | *IMV >2 days* | *29 (8.6)* | *46 (23)* | *22 (8.1)* | *1 (0)* |
|  | CRRT | 4 (1.2) | 18 (9) | 1 (0) | 0 (0) |
|  | ECMO | 6 (1.8) | 7 (3.5) | 0 (0) | 1 (0) |
|  | Inotropic support | 25 (7.4) | 43 (21.5) | 22 (8.1) | 0 (0) |

*Refers to 1^st^ admission of a patient; ^#^median (IQR); PICU = paediatric intensive care unit; † at admission; HSCT = hematopoietic stem cell transplantation; SOT = solid organ transplantation; IMV = invasive mechanical ventilation; CRRT = continuous renal replacement therapy; ECMO = extracorporeal membrane oxygenation.
