# Supplementary material for: Mortality of Hemato-Oncologic Patients Admitted to a Pediatric Intensive Care Unit: A Single-Center Experience
Source: Front Pediatr. 2022 Jul 12;10:795158. doi: 10.3389/fped.2022.795158 (PMC9315049; doi:10.3389/fped.2022.795158)
Supplement: Supplementary Table S2 — Admission mortality according to reason for emergency admission (N = 200). [file Table_2.DOCX]

**Supplemental Table 2**: Admission mortality according to reason for emergency admission (N=200)

|  | **Adm (% of N)** | **Died on PICU** | **Mortality %** |
| --- | --- | --- | --- |
| Respiratory disease | 73 (36.5) | 8 | 11 |
| Sepsis | 40 (20) | 6 | 15 |
| *with severe neutropenia* | *32 (16)* | *6* | *18.8* |
| *w/o severe neutropenia* | *8 (4)* | *0* | *0* |
| Neurologic injury | 31 (15.5.) | 2 | 6.5 |
| Acute GvHD | 10 (5) | 3 | 30 |
| Acute kidney injury | 9 (4.5) | 1 | 11.1 |
| Hyperleukocytosis | 8 (4) | 0 | 0 |
| Cardiovascular failure | 7 (3.5) | 1 | 14.3 |
| Febrile neutropenia w/o sepsis | 6 (3) | 1 | 16.7 |
| Electrolyte imbalance | 6 (3) | 0 | 0 |
| Other | 10 (5) | 0 | 0 |
| **Total** | **200** | **22** | **11** |

GvHD = Graft versus Host Disease; Other: gastrointestinal failure (n=4), anaphylaxis (n=3), haemolysis (n=2), severe hypoglycaemia (n=1); adm = admission(s); w/o = without.
