# Supplementary material for: Mortality of Hemato-Oncologic Patients Admitted to a Pediatric Intensive Care Unit: A Single-Center Experience
Source: Front Pediatr. 2022 Jul 12;10:795158. doi: 10.3389/fped.2022.795158 (PMC9315049; doi:10.3389/fped.2022.795158)
Supplement: Supplementary Table S3 — PICU mortality according to the number of organ system failures. [file Table_3.DOCX]

**Supplemental Table 3:** PICU mortality according to the number of organ system failures.

| **NOF** | **Admissions** | **Died in PICU** | **Mortality %** |
| --- | --- | --- | --- |
| 0 | 56 | 0 | 0 |
| 1 | 71 | 1 | 1.4 |
| 2 | 37 | 3 | 8.1 |
| >2 (MODS) | 36 | 18 | 50 |

NOF = Number of organ system failures; MODS = multiple organ dysfunction syndrome; PICU = paediatric intensive care unit.
