# Supplementary material for: Mortality of Hemato-Oncologic Patients Admitted to a Pediatric Intensive Care Unit: A Single-Center Experience
Source: Front Pediatr. 2022 Jul 12;10:795158. doi: 10.3389/fped.2022.795158 (PMC9315049; doi:10.3389/fped.2022.795158)
Supplement: Supplementary Table S4 — PICU mortality and inotropic support. [file Table_4.DOCX]

**Supplemental Table 4:** PICU mortality and inotropic support

| **Maximum number of Inotropic Agents during PICU stay** | **Admissions** | **Died at PICU** | **Admission Mortality %** |
| --- | --- | --- | --- |
| 0 | 157 | 7 | 4.5 |
| 1 | 17 | 1 | 5.9 |
| 2 | 9 | 2 | 22.2 |
| >2 | 16 | 11 | 68.8 |
